# Tratamiento actual de la enfermedad pulmonar intersticial asociada a la artritis reumatoide

**DOI:** 10.1016/j.opresp.2021.100106

**Published:** 2021-05-29

**Authors:** Alejandro Robles-Pérez, María Molina-Molina, Javier Narváez

**Affiliations:** a Servicio de Neumología, Hospital de Mataró – Consorci Sanitari del Maresme; b Unidad Funcional de Intersticio Pulmonar, Servicio de Neumología, Hospital Universitari de Bellvitge; c Servicio de Reumatología, Hospital Universitari de Bellvitge

La enfermedad pulmonar intersticial difusa (EPID) es la manifestación pulmonar más frecuente de la artritis reumatoide (AR) y actualmente representa la segunda causa de muerte en esta enfermedad^1^. A pesar de esta dramática situación, muchos son aún los aspectos que quedan por dilucidar en cuanto a su fisiopatología, cronología y, lo que es aún más apremiante, opciones de tratamiento.

El manejo de esta patología ha sido de creciente interés en los últimos años. Así lo demuestra el aumento del número de estudios llevados a cabo sobre la eficacia de tratamientos, principalmente con fármacos biológicos y antifibróticos.

A falta de unas guías de tratamiento, las estrategias terapéuticas en EPID-AR pueden variar de un centro a otro y se basan en series de casos o en la evidencia existente en otras enfermedades autoinmunes sistémicas (EAS), principalmente la esclerosis sistémica. Esta extrapolación no debería ser la norma, ya que existen importantes diferencias fisiopatológicas entre las diferentes EAS que pueden hacer variable la respuesta a un mismo tratamiento. Más recientemente la eficacia del tratamiento antifibrótico con nintedanib ha sido evaluado en un ensayo clínico que incluía pacientes con EPID fibrosantes y progresivas, siendo la EPID-AR la segunda entidad con más representación, con más del 10% del total de la muestra.

Contrariamente a teorías previas, a día de hoy existe evidencia sobre la seguridad a nivel pulmonar de los fármacos modificadores de la enfermedad (FAME), tales como metotrexato o leflunomida^2^, ^3^. Existen incluso trabajos que muestran un potencial efecto beneficioso sobre el pulmón, apoyando la idea de que un mejor control de la actividad sistémica puede contribuir a una mejor evolución de la afectación pulmonar^4^, ^5^.

Respecto al tratamiento de la afectación pulmonar y a pesar de la escasa evidencia existente, dosis variables de corticoides sistémicos, solos o en combinación con otros agentes inmunosupresores, constituyen aún a día de hoy el pilar de tratamiento en muchas ocasiones^6^. De entre los diferentes inmunosupresores disponibles actualmente, micofenolato mofetilo es el que ha presentado efectos más beneficiosos con un buen perfil de seguridad^7^.

Las terapias biológicas han mostrado en los últimos años una creciente evidencia de beneficio en la EPID-AR. De entre ellas, las mejor posicionadas son abatacept y rituximab. Abatacept, un inhibidor de la activación de linfocitos T, ha mostrado estabilidad o mejoría en pacientes con EPID-AR, medida mediante pruebas de función respiratoria (PFR) o tomografía computerizada de alta resolución (TCAR), entre un 85-91% de los casos en diferentes series^8^, ^9^. Recientemente se ha publicado la mayor serie de pacientes con EPID-AR evaluando la respuesta terapéutica a un fármaco, en este caso abatacept. Este trabajo, que incluye 263 pacientes, muestra ausencia de progresión en: disnea (91.9%), afectación en la TCAR (76.6%), capacidad vital forzada (FVC) (87.7%) y capacidad de difusión de mónoxido de Carbono (DLCO) (90.6%) en el seguimiento a 12 meses^10^.

En lo referente a rituximab, la depleción de linfocitos B CD20+ producida por el fármaco ha mostrado mejoría o estabilidad de la EPID-AR, medida mediante PFR o TCAR, en un 68-90% de casos^11^. Un reciente trabajo en 31 pacientes con EPID-AR fibrosante y progresiva en el año previo al inicio del fármaco ha mostrado una mejoría significativa de FVC (+8% y +11.2%) y de DLCO (+12.7% y +14.8%) al cabo de 1 y 2 años respectivamente del inicio de rituximab. En los primeros 12 meses un 94% de pacientes presentó ausencia de progresión^12^.

A pesar de estos resultados favorables existe un subgrupo de pacientes que presenta progresión tras recibir los diferentes tratamientos comentados. Es sabido que la AR asocia con mayor frecuencia que otras EAS un patrón de neumonía intersticial usual (NIU), presentando así similitudes clínicas y radiológicas con la fibrosis pulmonar idiopática (FPI). En este sentido y de forma similar a lo que sucede en la FPI, la respuesta terapéutica a antiinflamatorios e inmunosupresores en pacientes con NIU-AR es inferior a la de aquellos con patrón no NIU^11^.

En este aspecto el tratamiento antifibrótico con nintedanib se presenta como una opción terapéutica a tener en cuenta. Los resultados del análisis de subgrupos del estudio INBUILD mostraron que los pacientes con EPID fibrosante y progresiva asociada a EAS, de los cuales la gran mayoría eran pacientes con EPID-AR, tratados con nintedanib presentaron un descenso de la FVC 104 mL/año menor que aquellos tratados con placebo^13^. A pesar de que el diseño del estudio no estaba realizado para el análisis de subgrupos estos resultados arrojan esperanza para este grupo de pacientes. Recientemente se han publicado datos en práctica clínica habitual con nintedanib como uso compasivo en 7 pacientes con NIU-AR progresiva a los cuales se añadía el fármaco antifibrótico al tratamiento recibido hasta entonces (incluyendo corticoides, FAME, rituximab y abatacept). Los 6 pacientes que completaron más de 6 meses de tratamiento presentaron estabilización de la afectación pulmonar que hasta el inicio del fármaco era progresiva. El perfil de seguridad fue similar al de estudios previos, sin existir ningún caso de infección severa, a pesar de la asociación con FAMEs o rituximab^14^.

El trasplante pulmonar es una opción en casos de enfermedad severa y progresiva. A pesar de que la presencia de una EAS pueda parecer de entrada un factor de riesgo de complicaciones extrapulmonares post-trasplante, un estudio retrospectivo mostró una supervivencia a 1 año similar en pacientes con FPI en comparación con pacientes con EPID-AR^15^.

El tratamiento de la EPID-AR constituye actualmente un reto para el clínico tratante, especialmente en pacientes con enfermedad progresiva. En estos casos un buen control de la actividad inflamatoria sistémica, que podría actuar como desencadenante del daño pulmonar, así como una inhibición del proceso fibrogénico a nivel pulmonar constituyen a día de hoy las estrategias más prometedoras en EPID-AR. El beneficio de la asociación de tratamiento inmunosupresor y antifibrótico podría suponer una opción terapéutica eficaz y su beneficio y seguridad deberían ser evaluados en futuros estudios.

## Financiación

Los autores no han contado con financiación para la elaboración del presente artículo.

## Conflicto de interés

Los autores declaran no tener conflictos de interés en relación al contenido del presente trabajo.
